# Aggregation-Enhanced Room-Temperature Phosphorescence from Au(I) Complexes Bearing Mesogenic Biphenylethynyl Ligands

**DOI:** 10.3390/molecules26237255

**Published:** 2021-11-30

**Authors:** Andriani Furoida, Misato Daitani, Kyohei Hisano, Osamu Tsutsumi

**Affiliations:** Department of Applied Chemistry, Ritsumeikan University, 1-1-1 Nojihigashi, Kusatsu 525-8577, Japan; gr0450vs@ed.ritsumei.ac.jp (A.F.); d.misa3310@gmail.com (M.D.); hisano@fc.ritsumei.ac.jp (K.H.)

**Keywords:** aggregation-induced emission, gold(I) complex, aurophilic interaction, liquid crystal, aggregated structure

## Abstract

Gold(I) complexes, enabling to form linear coordination geometry, are promising materials for manifesting both aggregation-induced emission (AIE) behavior due to strong intermolecular Au–Au (aurophilic) interactions and liquid crystalline (LC) nature depending on molecular geometry. In this study, we synthesized several gold(I) complexes with rod-like molecular skeletons where we employed a mesogenic biphenylethynyl ligand and an isocyanide ligand with flexible alkoxyl or alkyl chains. The AIE behavior and LC nature were investigated experimentally and computationally. All synthesized gold(I) complexes exhibited AIE properties and, in crystal, room-temperature phosphorescence (RTP) with a relatively high quantum yields of greater than 23% even in air. We have demonstrated that such strong RTP are drastically changed depending on the crystal-size and/or crystal growth process that changes quality of crystals as well as the aggregate structure, of e.g., Au–Au distance. Moreover, the complex with longer flexible chains showed LC nature where RTP can be observed. We expect these rod-like gold(I) complexes to have great potential in AIE-active LC phosphorescent applications such as linearly/circularly polarizing phosphorescence materials.

## 1. Introduction

Luminescent organic materials generally only exhibit efficient luminescence in dilute solutions, which is diminished once they form aggregates. This decrease in the luminescence intensity is known as the aggregation-caused quenching (ACQ) effect, which is the principal concern for practical luminescent applications [1]. In 2001, Tang et al. introduced aggregation-induced emission (AIE) phenomena where, in contrast to ACQ, the aggregation of molecules significantly enhanced the emission [2]. Many types of organic AIE-active molecules have been reported to have potential in light-emitting applications, such as cell imaging, chemo-sensors, biosensors, and organic light-emitting diodes.

Gold(I) complexes have been recognized as a type of AIE material, as their luminescence is significantly enhanced in the solid-state. Au–Au (aurophilic) interactions play an important role in inducing efficient luminescence from aggregates. This interaction is expressed when the distance between neighboring gold atoms falls in the range of 2.8–3.6 Å [3,4,5,6,7,8,9,10,11,12,13]. Since the emission from gold(I) complexes originate mostly from aggregates, their photophysical properties, such as emission color and intensity, are highly sensitive to changes in the aggregated structure. Significant efforts have been made to study the unique properties of aurophilic systems and to control their photophysical properties using external stimuli, such as heat, pH, and mechanical force [10,11,12]. In particular, liquid-crystalline (LC) gold(I) complexes have attracted much attention in controlling the aggregated structure due to the nature of LC to both self-organize and be responsive to external fields (e.g., electric and magnetic fields). Therefore, AIE-active LC gold(I) complexes have great potential because their aggregated structures can be readily controlled by external stimuli.

**Scheme 1:**
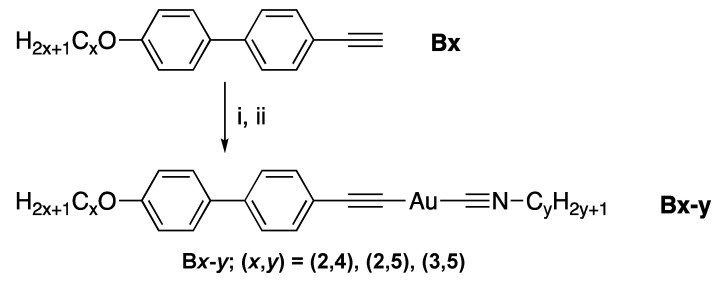
Synthetic route for gold(I) complexes **B*x***-***y***. (i) (tht)AuCl/CH_2_Cl_2_, sodium acetate/MeOH, (ii) C≡N–C*_y_*H_2*y* + 1_. Isolated yields of complexes: 28% (**B2-4**), 18% (**B2-5**), and 50% (**B3-5**).

The molecular skeleton of LCs generally consists of both a rod-like rigid core and a flexible chain consisting of alkoxy and alkyl chains. Rod-like gold(I) complexes have been synthesized, leading to the development of various AIE-active LC systems [3,8]. Recently, we reported a series of gold(I) complexes synthesized using ligands with less steric hindrance around the Au atoms to manifest aurophilic interactions. In particular, our synthesized gold(I) complex with both an isocyanide and a phenylacetylene ligand exhibited interesting AIE properties. In addition, the materials showed very high room-temperature phosphorescence (RTP) quantum yields (>50%) in crystals that is developed from an extremely efficient S_0_–T*_n_* direct transition [8]. This complex showed an LC feature at approximately 90 °C, although the LC phase had low thermodynamic stability and weak luminescence. In this work, we investigated the effect of the molecular aspect ratio, which significantly affects the LC nature. We designed rod-like gold(I) complexes with a biphenyl acetylene and isocyanide ligand, as shown in Scheme 1, referred to as **B*x*-*y***, where *x* represents the length of the alkoxy chain incorporated into the biphenyl structure and *y* represents the length of the alkyl chain of the isocyanide group. We expect that by incorporating biphenyl acetylene ligands with longer flexible chains, instead of the phenylacetylene derivatives, may enable the expansion of the LC temperature range. Additionally, restricting intramolecular movements of the biphenyl rings may enhance the luminescence intensity even in the LC phase. Furthermore, we investigated the effect of the length of the alkoxy and alkyl chains of the complexes on both the AIE properties and LC behavior. We found that the length of the flexible chains greatly affects the LC properties of rod-like gold(I) complexes, since only **B3-5** shows the LC phase. All gold(I) complexes exhibited AIE, and the luminescence was observed in both the crystal and the LC phase.

## 2. Results and Discussion

### 2.1. Synthesis and Characterization of Gold Complexes

The rod-like gold(I) complexes, **B*x*-*y***, were obtained from the corresponding **B*x*** ligands (Scheme 1). These complexes were successfully purified by column chromatography using silica gel, followed by recrystallization from a mixed solvent system of CH_2_Cl_2_ and hexane. The complexes were fully characterized by ^1^H nuclear magnetic resonance (NMR) spectroscopy, infrared (IR) spectroscopy, and elemental analysis. All analytical data (presented in the Experimental section) confirmed that the desired complexes were obtained.

**Figure 1:**
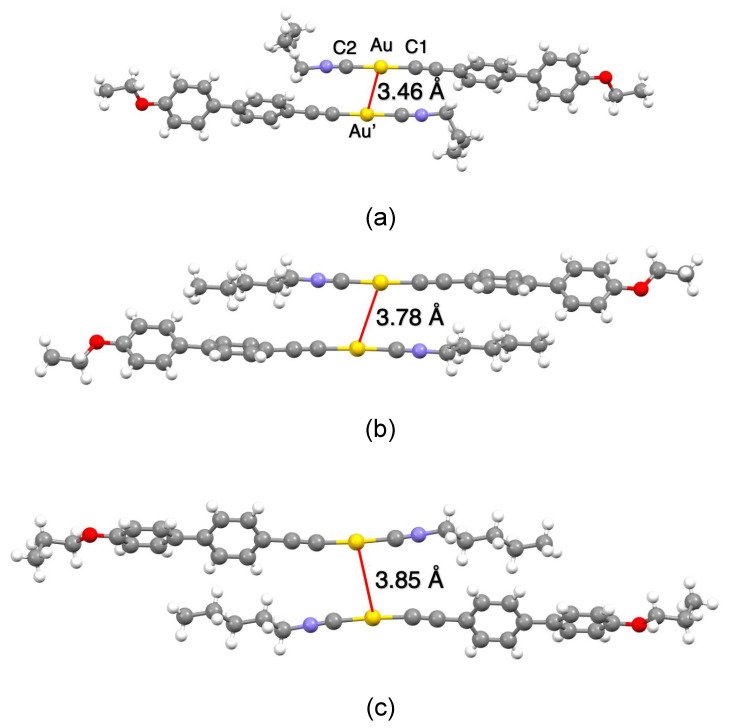
Crystal packing structure of (**a**) **B2-4,** (**b**) **B2-5,** and (**c**) **B3-5** at room temperature. Red lines represent the Au–Au distances. Atom color legends: gray, C; blue, N; red, O; yellow, Au; white, H.

To elucidate the molecular structures of the complexes in the crystal, we conducted single-crystal X-ray diffraction (XRD) structural analysis at room temperature. Figure 1 shows the crystal packing structures of the complexes, where the selected interatomic distances between the closest neighboring molecules are depicted. Selected bond angles of the complexes are listed in Table 1, and the key crystallographic data are listed in Table S1. According to the crystal structures, all complexes were arranged in an anti-parallel orientation with the Au–Au distance between the closest neighboring molecules ranging between 3.4 and 3.8 Å. Considering that the Au–Au distance in the **B2-4** crystal (3.46 Å) is less than the sum of the van der Waals radii of two gold atoms (3.6 Å) [14,15], the **B2-4** complex forms an aurophilic interaction. The **B2-5** and **B3-5** complexes have an Au–Au distance slightly greater than 3.6 Å, although some previous reports suggest that the sum of the van der Waals radii is not the only indicator of aurophilic interactions [16]. We found that all synthesized gold(I) complexes possess a slightly bent C1–Au–C2 bonding angle (Figure 1 and Table 1), as opposed to the general C1–Au–C2 bonding angle of 180° in typical gold(I) complexes [17]. This suggests that the strong Au–Au interactions in the present complexes distorts the bonding angle, which decreases the distance between the Au atoms in the crystal.

We evaluated the thermal stability of the gold complexes using a thermogravimetric/differential thermal analyzer (TG-DTA) at a scanning rate of 5 °C min^−1^ from 25 to 600 °C. The TG-DTA thermograms are shown in Figure S4. The thermal decomposition temperature (*T*_dec_) is defined as the temperature when a 5% weight loss of compounds occurs during heating in air [7,8,9]. As summarized in Table 2, the *T*_dec_ values of all gold(I) complexes were approximately 180 °C. Around *T*_dec_, a 16% weight loss was observed for **B2-4** and **B2-5**, and 18% for **B3-5**, corresponding to the isocyanide moieties. As reported previously, this result suggests that the thermal decomposition of gold(I) complexes with isocyanide ligands is initiated by bond cleavage between Au and the isocyanide ligand [8]. The second weight loss of approximately 26% occurred in all complexes after heating to 400 °C, which corresponds to the cleavage of the alkoxy ligand. The residual ash obtained after heating to 600 °C corresponded with the calculated percentage of gold atoms (~38%); thus, the remaining residue after heating was confirmed to be gold ash. This result is also consistent with the result of the elemental analysis for those complexes (see Section 3).

**Figure 2:**
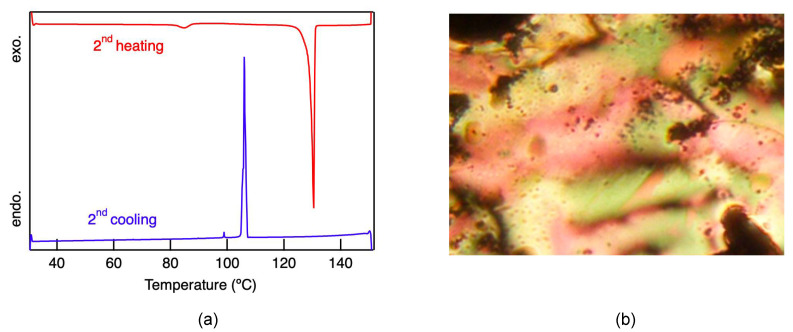
(**a**) A DSC thermogram (second scan) of **B3-5**: scanning rate = 2 °C min^−1^; red line, heating; blue line, cooling. (**b**) A POM image of **B3-5**, showing the N phase, observed at 140 °C.

We analyzed the phase transition behavior of gold(I) complexes by differential scanning calorimetry (DSC) measurements and polarized optical microscopy (POM) observations, as summarized in Table 2. **B2-4** and **B2-5** did not exhibit an LC phase at any temperature. Both **B2-4** and **B2-5** showed crystal-to-isotropic phase transition. In **B2-4**, after melting at 123 °C in the first heating process, no peak was observed in both cooling and heating process in the DSC thermogram due to supercooling (Figure S5). In contrast, the reversible crystal-to-isotropic phase transition was observed by DSC in complex **B2-5** (Figure S6). Moreover, **B3-5** showed enantiotropic LC behavior, indicated by intense exo- and endothermic peaks appearing during both the heating and cooling process, as shown in Figure 2a. These results are in good agreement with that previously reported for LC compounds. Additionally, in our gold(I) complex, the longer flexible chains on the same rigid core unit exhibits a lower melting point and/or LC properties. To further investigate the LC properties of **B3-5**, we conducted detailed POM measurements, as shown in Figure 2b. A schlieren texture was observed at 140 °C, indicating that the observed LC phase in **B3-5** is N (Figure 2b). Compared with the previously reported Au complex bearing phenylacetylene ligands, complex **B3-5** exhibited N phase in a wider temperature range, meaning that the N phase in complex **B3-5** is thermodynamically stable [8]. In the DSC thermogram of **B3-5**, a small endothermic peak was observed at 80 °C during the heating process. However, during the cooling process, a small exothermic peak appeared at 102 °C. However, no change in the optical texture was observed by POM at these temperatures. Therefore, we conclude that **B3-5** showed polymorphism in the crystal phase, and these small exo- and endothermic peaks were assigned to the crystal-to-crystal phase transition. 

### 2.2. Photoluminescence Properties of Gold Complexes

**Figure 3:**
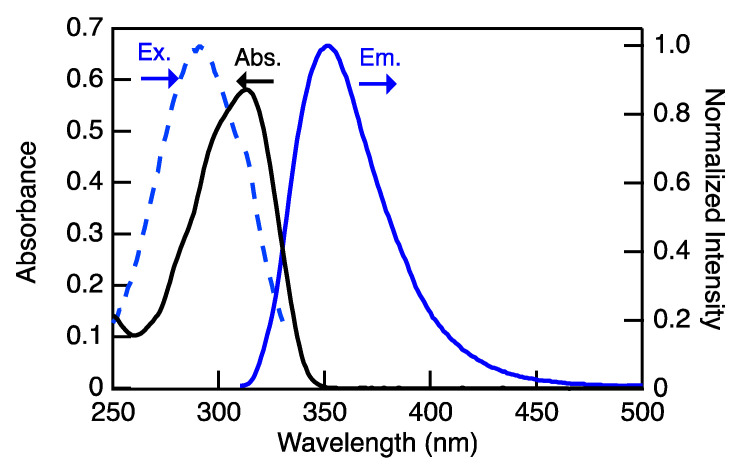
Photophysical properties of **B2-5** in a CH_2_Cl_2_ solution: absorption, solid black line; emission, solid blue line; excitation, dashed blue line. [**B2-5**] = 1 × 10^−5^ mol L^−1^ for absorption spectra; [**B2-5**] = 1.5 × 10^−6^ mol L^−1^ for luminescence spectra. The excitation wavelength (*λ*_ex_) was 291 nm. Excitation spectra were monitored at 348 nm.

UV-vis absorption and photoluminescence spectra in a dilute CH_2_Cl_2_ solution are shown in Figure 3 for **B2-5** as a typical example ([**B2-5**] was 1 × 10^−5^ and 1.5 × 10^−6^ mol L^−1^ for the absorption and luminescence spectra, respectively). Dilute solutions of the gold(I) complexes absorbed UV light at 313 nm with a molar extinction coefficient (*ε*) of 5.8 × 10^4^ L mol^−1^ cm^−1^, attributed to the π–π* transition of the ligand [18,19]. Upon excitation at 293 nm, the dilute solution showed a weak emission band at 350 nm when measured in air, corresponding to luminescence from the isolated molecule (monomer emission). The excitation band was slightly blue-shifted compared to the absorption band. We consider that this difference resulted from the formation of aggregates, which is much more emissive than the monomer of the complex [8]. Complexes **B2-4** and **B3-5** showed similar behaviors to the **B2-5** complex in solution.

**Figure 4:**
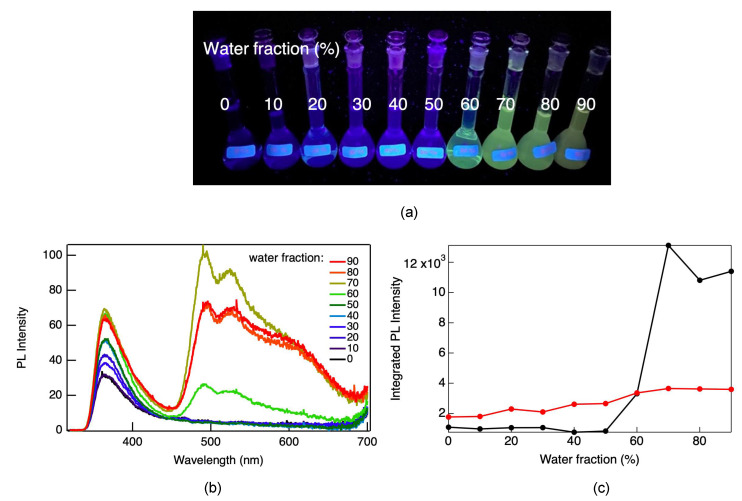
(**a**) Photograph of **B2-5** in THF/water mixed solvent (*f*_w_ = 0–90%) under a 365 nm UV light, (**b**) Emission spectra of **B2-5** in *f*_w_ = 0–90% with a concentration of 1 × 10^−^^4^ mol L^−^^1^ (*λ*_ex_ = 303 nm). (**c**) Integrated photoluminescence intensity in the range of 330–450 nm (red) and 450–700 nm (black) as a function of *f*_w_.

As a representative example, we investigated the AIE activity of **B2-5** by observing the luminescence behavior in a THF/water mixed solvent system (Figure 4). In pure THF, the complex showed weak luminescence at ~360 nm, similar to the dilute CH_2_Cl_2_ solution; thus, this emission band can be assigned to the monomer emission. However, as shown in Figure 4b, **B2-5** showed dual emissions at 360 and 500 nm in the THF/water mixed solvent. Increasing the water fraction (*f*_w_) increased the intensity of the shorter-wavelength luminescence band gradually. Owing to the increasing fraction of the poor solvent (water), the complex formed aggregates in the mixed solvent. Addition of a poor solvent significantly enhanced the total intensity of luminescence, signifying that the synthesized gold(I) complex of **B2-5** is an AIE-active material [20,21,22,23]. At a *f*_w_ > 80%, the total luminescence intensity decreased due to the precipitation of relatively large crystals, indicating that the phenomenon was not induced by the ACQ effect.

In contrast to the shorter-wavelength band showing a gradual increase in intensity, the luminescence intensity of the longer-wavelength band at 500 nm increased abruptly at *f*_w_ = 60%, followed by a distinct change in the luminescence color from blue to yellow (Figure 4a). This luminescence band was only observed in the mixed solvent with a *f*_w_ > 60%, wherein the complex formed large aggregates. This phenomenon is attributed to the size-dependent crystalline polymorphism and crystal-to-crystal phase transition induced during the crystal growth process of gold(I) complexes in the mixed solvent. We previously reported that in some gold(I) complexes, the stability of the crystal structure depends on the crystal size; thus, in the crystal growth process, the crystal-to-crystal phase transition occurs at the critical size (several micrometers) [24]. This phase transition resulted in a reversible RTP color change. Therefore, the synthesized gold(I) complexes of **B2-5** exhibited similar phenomena to the previously reported system; that is, (1) small crystals (or crystal nuclei), which were initially formed by adding water, showed monomeric luminescence at ~360 nm, and (2) crystals grew to the critical size at *f*_w_ > 60% where the crystal-to-crystal phase transition occurred. In crystals larger than the critical size, the molecules form dimers that show emission at ~500 nm due to aurophilic interactions. Similar results were observed in the AIE experiment for the **B3-5** complex (Figure S9).

**Figure 5:**
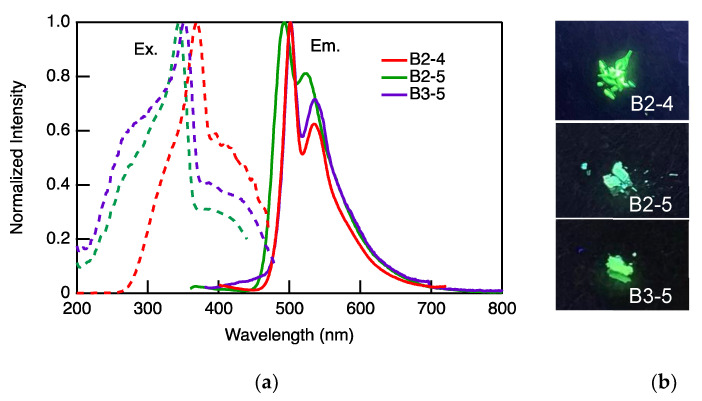
(**a**) Excitation (dashed line) and emission spectra (solid line) of gold(I) complexes in the crystal. The *λ*_ex_ for emission spectra were shown in Table 3. Excitation spectra were obtained at the maximum wavelength for emission (*λ*_max_^lum^) shown in Table 3. Red, **B2-4**; green, **B2-5**; purple, **B3-5**. (**b**) Photographs of gold(I) complexes taken under 365 nm UV irradiation.

To confirm this hypothesis, we investigated the luminescence behavior of the complexes in the bulk crystals prepared by recrystallization. All gold(I) complexes reported in this paper emitted intense luminescence at approximately 500 nm in the crystal with a relatively high *Φ* of 23% (Figure 5 and Table 3). Compared with the luminescence spectra in the dilute solutions, the luminescence band in the crystal was considerably red-shifted, suggesting that the emission arises from a lower energy excited state, such as the triplet state [25,26]. The same luminescence band at 500 nm was also observed in the degassed dilute solution (in THF, 10^−6^ mol L^−1^), which supports that the emission corresponds to a phosphorescence band (Figure S10). However, in the solution, the relative intensity of the longer-wavelength luminescence was much weaker than that of the monomer emission at 360 nm. In contrast, the crystals exhibited very intense luminescence at 500 nm and almost no luminescence at ~400 nm, indicating that the luminescence band at 500 nm originates from the aggregates of the complexes. This luminescence band in the crystals was consistent with that observed at the same wavelength in the mixed solvent (Figure 4). Therefore, these results support our hypothesis that the luminescent aggregates formed during the crystal growth process in the mixed solvent by the crystal–crystal phase transition at the critical crystal size.

Crystals of all the complexes under investigation exhibited strong luminescence (~500 nm) with the same spectral shape at almost the same wavelength, which suggests that the molecules formed luminescent aggregates with the same structure in the crystal, consistent with the crystallographic results (Table S1).

To obtain further information about the photophysical properties of the complexes, photoluminescence lifetime (*τ*) and quantum yields (*Φ*) were measured in the crystalline phase (Table 3). Biexponential decay profiles with lifetimes in the order of microseconds were observed in the crystals. From the lifetime, we conclude that the observed luminescence in the crystal at ~500 nm is phosphorescence. Nevertheless, high *Φ* values were obtained for all complexes (up to 23%). It is generally difficult to obtain phosphorescence emission with high *Φ* under ambient conditions because the triplet excited state is easily deactivated by molecular oxygen and thermal motion of molecules. This intense RTP is attributed to crystallization-induced phosphorescence, because the densely packed crystals inhibit the diffusion of molecular oxygen and molecular motions [27,28,29]. Furthermore, based on the *Φ* and *τ* values, the rate constants for radiative transition (*k*_r_) and non-radiative deactivation (*k*_nr_) of the complexes in the crystal were estimated. As summarized in Table 3, the *k*_nr_ values were similar in all complexes. Thus, the *Φ* values only depends on the *k*_r_.

In the excitation spectra of the crystals shown in Figure 5, an excitation band appeared at ~300 nm, assigned to the S_0_–S*_n_* transition [30], which was also observed in the absorption spectra of the solution (Figure 3). However, an additional excitation band was observed at ~450 nm in the crystals. We previously attributed this excitation band to a spin-forbidden S_0_–T*_n_* transition [30], which cannot generally be observed. However, this type of Au complex, with ethynyl and isocyanide ligands, shows an efficient S_0_–T*_n_* transition due to aggregation; that is, the S_0_–T*_n_* transition is not only enhanced by heavy atom effects, but also by the aggregation of Au complexes [30]. The present complexes showed large S_0_–T*_n_* transitions, which explains why the materials can show relatively large phosphorescence *Φ* in the crystals under ambient conditions.

**Figure 6:**
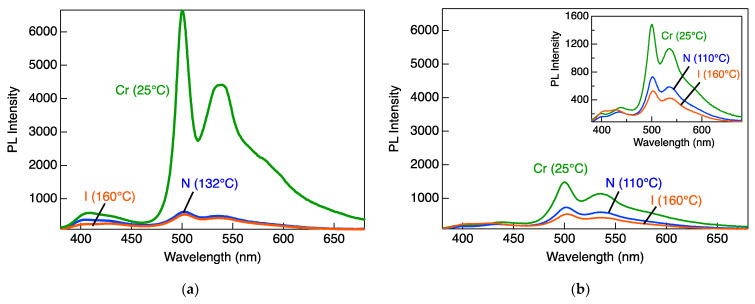
Photoluminescence spectra of **B3-5** (*λ*_ex_ = 352 nm) in various phases obtained from (**a**) heating the crystal and (**b**) freezing the molten states. Inset: magnification of emission spectra during the freezing process. Green, Cr; blue, N; orange, I.

The photoluminescence spectra of **B3-5** in the LC phase were also investigated to determine the effects of the phase structure on the luminescence behavior (Figure 6). The emission intensity of **B3-5** decreased in both the N and I phases (at a temperature higher than the crystal) owing to the activated thermal motion that promotes the non-radiative process. [7,8]. Interestingly, the present isocyanide complex has a higher luminescence efficiency in the LC phase than those with a phenylethynyl ligand, as previously reported [3]. In addition, the luminescence intensity was recovered in the crystal obtained after cooling the molten sample. However, the emission intensity of the frozen crystal was lower than that of the original crystal. This is attributed to the crystal-size dependency on the aggregation-enhanced spin-forbidden transition reported recently [30]. The aggregation-enhanced spin-forbidden transition originates from the Au–Au interactions formed in the crystal; thus, this effect depends on the quality as well as the size of the crystal. The size of the crystal obtained by cooling from the I phase was smaller than the original crystal obtained from the recrystallization, leading to a decrease in the *Φ* of RTP. These results suggest that the RTP properties of the Au complexes are sensitive to both the structure and size of the aggregates.

### 2.3. Computational Studies

**Figure 7:**
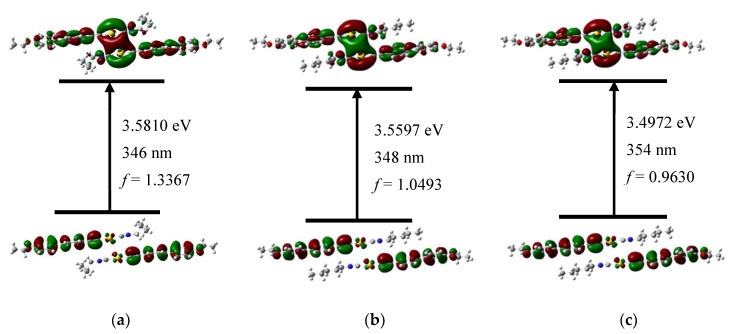
Molecular orbital (HOMO and LUMO), transition energy, excitation wavelength, and oscillator strength (*f*) of the gold(I) complexes (**a**) **B2-4**, (**b**) **B2-5**, and (**c**) **B3-5** obtained by TD-DFT calculations employing B3LYP hybrid functionals with SDD (for the Au atoms) and 6-311 + G(d,p) basis sets for all other atoms.

To further understand the photophysical properties of the complexes, the molecular orbitals were calculated using time-dependent density functional theory (TD-DFT) by employing B3LYP hybrid functionals with SDD (for the Au atoms) and 6-311 + G(d,p) (for the remaining atoms) basis sets. The calculations were performed for the dimer formed in the single crystal, and the oscillator strength (*f*) distribution calculated for the transitions to the singlet excited states is summarized in Table 4. For the calculated electron transitions with large *f* values at ~350 nm (*f* = ~1), the corresponding molecular orbitals are shown in Figure 7. DFT calculations revealed that the excitation band at ~350 nm corresponds to a transition from S_0_ to the lowest excited singlet state (S_1_), and the main contribution to this transition is a one-electron excitation process from the HOMO to the LUMO. From the electron distributions of the HOMO and LUMO states shown in Figure 7, this transition occurs from the π orbital of the ethynyl biphenyl to the Au–Au atoms and is thus a ligand-to-metal-metal charge transfer transition [28,30]. The *d*_Au–Au_ values of the **B2-5** and **B3-5** complexes are slightly higher than the sum of the van der Waals radii of the Au atoms; however, these computational results suggest that the absorption and luminescence of all the reported complexes are a result of the same electronic transition.

## 3. Materials and Methods

### 3.1. Preparation of Materials

Rod-like gold(I) complexes were prepared from the corresponding 4-alkoxybiphenylacetylene ligand (**B*x***), as shown in Scheme 1. **B*x*** ligand and tht(AuCl) (tht = tetrahydrothiophene) were synthesized by the method reported previously [8]. Unless otherwise noted, all solvents and reagents used were commercially available and were used without further purification. ^1^H NMR spectra were recorded using an ECS-400 spectrometer (JEOL, Tokyo, Japan) at 400 MHz. Chemical shifts are reported in parts per million (ppm), with the residual protons of the NMR solvent used as an internal reference. IR spectra were recorded on an FT/IR-4100 spectrometer (JASCO, Tokyo, Japan) using KBr pellets. Elemental analysis of C, H, and N atoms was performed using a MICRO CORDER JM10 analyzer (J-SCIENCE, Tokyo, Japan).

**B2-4**. 4-ethoxy-4′-ethyl-1,1′-biphenyl (**B2**, 0.15 g, 0.67 mmol) and (tht)AuCl (0.25 g, 0.13 mmol) were dissolved in 10 mL of CH_2_Cl_2_. A MeOH (7 mL) solution of CH_3_COONa (0.27 g, 3.4 mmol) was added dropwise to the **B2** solution and stirred for 2 h at room temperature. The residue was collected by filtration and washed with MeOH, H_2_O, and CH_2_Cl_2_. The obtained yellow solid was suspended in CH_2_Cl_2_, followed by the addition of 1-butyl-isocyanide (22 mg, 0.26 mmol) and stirred for 2 h at room temperature to give a light-yellow homogenous solution. The resultant **B2-4** solution was filtered using celite and purified by silica column chromatography (eluent: CH_2_Cl_2_). The obtained white solid was recrystallized from a mixed solution of CH_2_Cl_2_ and hexane, yielding yellow crystals of **B2-4** in 28% (29 mg) yield. mp 123 °C. ^1^H NMR (400 MHz, CDCl_3_, *δ*): 7.50–7.42 (m, 6H; 3′,5′-*H* in biphenyl, 2′,6′-*H* in biphenyl, 3,5-*H* in biphenyl), 6.93 (dd, *J* = 6.5, 2.0 Hz; 2H; 2,6-*H* in biphenyl), 4.06 (q, *J* = 6.9 Hz; 2H; OC*H*_2_), 3.63 (t, *J* = 6.8 Hz; 2H; NC*H*_2_), 1.81 (m, 2H; NCH_2_C*H*_2_), 1.54–1.41 (m, 5H; NCH_2_CH_2_C*H*_2_, OCH_2_C*H*_3_), 0.99 (t, *J* = 7.2 Hz; 3H; N(CH_2_)_3_C*H*_3_). FTIR (KBr, *ν*): 3024, 2972, 2251, 1245, 1043 cm^−1^. Anal. calcd. for C_21_H_22_AuNO: C, 50.31; H, 4.42; N, 2.79; Au, 39.28. Found: C, 50.13; H, 4.34; N, 2.77; Ash, 39.3.

**B2-5** and **B3-5.** These complexes were synthesized according to the same procedure used for **B2-4**, and the corresponding yields for **B2-5** and **B3-5** were 18% (25 mg) and 50% (75 mg), respectively.

**B2-5**. mp 139 °C. ^1^H NMR (400 MHz, CDCl_3_, *δ*): 7.51–7.42 (m, 6H; 3′,5′-*H* in biphenyl, 2′,6′-*H* in biphenyl, 3,5-*H* in biphenyl), 6.95 (dd, *J* = 6.5, 2.0 Hz; 2H; 2,6-*H* in biphenyl), 4.05 (q, *J* = 7.0 Hz; 2H; OC*H*_2_), 3.61 (t, *J* = 6.5 Hz; 2H; NC*H*_2_) 1.83 (m, 2H; NCH_2_C*H*_2_), 1.46–1.35 (m, 7H; NCH_2_CH_2_(C*H*_2_)_2_, OCH_2_C*H*_3_), 0.94 (t, *J* = 7.1 Hz; 3H; N(CH_2_)_4_C*H*_3_). FTIR (KBr, *ν*): 3021, 2945, 2251, 1240, 1048 cm^−^^1^. Anal. calcd. for C_22_H_24_AuNO: C, 51.27; H, 4.69; N, 2.72; Au, 38.22. Found: C, 51.20; H, 4.68; N, 2.79; Ash, 38.0.

**B3-5**. mp 132 °C. ^1^H NMR (400 MHz, CDCl_3_, *δ*): 7.49–7.41 (m, 6H; 3′,5′-*H* in biphenyl, 2′,6′-*H* in biphenyl, 3,5-*H* in biphenyl), 6.98 (dd, J = 6.5, 2.0 Hz; 2H; 2,6-*H* in biphenyl), 3.94 (t, *J* = 6.5 Hz; 2H; OC*H*_2_), 3.62 (t, *J* = 6.7 Hz; 2H; NC*H*_2_), 1.84–1.77 (m, 4H; OCH_2_C*H*_2_, NCH_2_C*H*_2_), 1.45–1.34 (m, 4H; NCH_2_CH_2_(C*H*_2_)_2_), 1.03 (t, *J* = 7.5 Hz; 3H; O(CH_2_)_2_C*H*_3_), 0.94 (t, *J* = 7.1 Hz, 3H; N(CH_2_)_4_C*H*_3_). FTIR (KBr, *ν*): 3022, 2251, 2922, 1241, 1045 cm^−^^1^. Anal. calcd. for C_23_H_26_AuNO: C, 52.18; H, 4.95; N, 2.65; Au, 37.20. Found: C, 51.88; H, 4.84; N, 2.73; Ash, 36.8.

### 3.2. Single-Crystal XRD Analysis

Single crystals were prepared by slow evaporation from a mixed solvent system (hexane and CH_2_Cl_2_). Each crystal was mounted on a glass fiber, and the reflection data were collected with the omega scanning technique using a D8 goniometer (Bruker, Billerica, MA, USA). Monochromatic Mo *K*α radiation (*λ* = 0.71075 Å) was used to analyze the complexes and all measurements were performed at room temperature (298 K). The initial structures of all the complexes were determined using a direct method using APEX3. The structural model was refined by a full-matrix least-squares method using SHELXL-2014/6. All calculations were conducted using SHELXL programs. The crystal data are summarized in Table S1, and are included in the Cambridge Crystallographic Data Centre (CCDC) database as reference numbers CCDC: 2,123,806 for **B2-4**, 2,123,807 for **B2-5**, and 2,123,809 for **B3-5**, respectively. The indexed database contains additional supplementary crystallographic data for this paper and may be accessed without charge at http://www.ccdc.cam.ac.uk/conts/retrieving.html (accessed on 6 November 2021).

### 3.3. Thermal Stability and Phase Transition Behavior Analysis

The thermal stability of the gold(I) complexes was evaluated using TG-DTA (DTG-60AH, Shimadzu, Kyoto Japan) with α-alumina at a heating rate of 5 °C min^−1^. The phase transition behavior of gold(I) complexes was observed using DSC (X-DSC7000, SII) and POM (BX51, Olympus, Tokyo, Japan) at a scanning rate of 5 °C min^−1^ for **B2-4** and **B2-5** and 2 °C min^−1^ for **B3-5**.

### 3.4. Photophysical Properties

UV-visible absorption and steady-state photoluminescence spectra were recorded using an absorption spectrophotometer V-550 (JASCO, Tokyo, Japan) and a fluorescence spectrophotometer F-7500 (Hitachi, Tokyo, Japan), respectively. The photoluminescence quantum yield (*Φ*) was measured using a calibrated integration sphere system (Hitachi, Tokyo, Japan). Photoluminescence decay profiles were recorded using a Quantaurus-Tau photoluminescence lifetime (*τ*) measurement system C1136-21 (Hamamatsu Photonics, Hamamatsu, Japan).

### 3.5. Computational Studies

TD-DFT calculations were conducted using the Gaussian 16 (revision C.01, Gaussian, Inc., Wallingford, CT, USA) program package, employing the B3LYP hybrid functional with SDD and 6-311G + (d,p) basis sets for Au and the remaining atoms, respectively. Single-point calculations were conducted using the conformation determined by single-crystal structure analysis.

## 4. Conclusions

In this study, we synthesized a series of gold(I) complexes with a rod-like molecular skeleton with both a biphenylethynyl ligand and an isocyanide ligand. All synthesized gold(I) complexes exhibited AIE properties and RTP in the crystal state with the *Φ* greater than 23% even in air. Such strong RTP is attributed to the aggregation-enhanced spin-forbidden S_0_–T*_n_* transition arising not only from the heavy atom effect of Au atoms but also from Au–Au interaction in the densely packed crystals. Interestingly, the gold(I) complexes showed crystal-size dependent polymorph and crystal-to-crystal phase transition during crystal growth process; and thus, the luminescence color and *Φ* were significantly affected by the crystal quality. Furthermore, the complex of **B3-5** with the longer flexible chains incorporated into the rigid rod-like units showed LC behavior. The LC feature in **B3-5** complex possesses relatively high thermodynamic stability and luminescence efficiency rather than our previously synthesized compounds, which might be due to the larger molecular aspect ratio. Such AIE-active LC of **B3-5** is expected to be used as molecularly ordered phosphorescent AIEgens with a great potential in the field of linearly and/or circularly polarized phosphorescent applications.

## Figures and Tables

**Table 1:** Au–Au distances between the neighboring molecules and selected bond angles in **B*x*-*y*** crystals.

Complex	Au–Au’ (Å)	C1–Au–C2 (°)
**B2-4**	3.46	175.8
**B2-5**	3.78	176.1
**B3-5**	3.85	174.8

**Table 2:** Thermo-chemical properties and phase transition behaviors of **B*x*-*y*** complexes.

Complex	*T*_dec_ (°C)	Phase Transition Temperature (°C)
**B2-4**	176	Cr 123 I
**B2-5**	183	Cr 139 I
**B3-5**	183	Cr_1_ 80 Cr_2_ 132 N 160 I

Abbreviations: *T*_dec_, thermal decomposition temperature; Cr, crystal; N, nematic; I, isotropic.

**Table 3:** Photophysical parameters of gold(I) complexes in the crystal at room temperature.

Complex	*λ*_ex_ (nm)	*λ*_max_^lum^ (nm)	*Φ* (%)	*τ* (µs)	*k*_r_ (s^−1^) ^a^	*k*_nr_ (s^−1^) ^b^
**B2-4**	368	500	21	21 (64%), 150 (36%)	3.0 × 10^3^	1.1 × 10^4^
**B2-5**	344	492	14	36 (81%), 363 (19%)	1.9 × 10^3^	1.1 × 10^4^
**B3-5**	352	500	23	23 (78%), 178 (22%)	3.8 × 10^3^	1.4 × 10^4^

^a^*k*_r_ is estimated by *Φ/τ*; ^b^ *k*_nr_ is estimated as (1−*Φ*)*/τ*. The averaged *τ* of the biexponential decay was used for calculating *k*_r_ and *k*_nr_ [9,27].

**Table 4:** TD-DFT results for all gold complexes.

Complex	*d*_Au–Au_ (Å)	First Excitation Energy	*f*
**B2-4**	3.46	3.58 eV (346 nm)	1.34
**B2-5**	3.78	3.55 eV (348 nm)	1.04
**B3-5**	3.85	3.49 eV (354 nm)	0.96

## Data Availability

The authors confirm that the data supporting the findings of this study are available within the article and Supplementary Materials.

## Supplementary Materials

The following are available online, Figure S1: ^1^H NMR spectrum of **B2-4**, Figure S2: ^1^H NMR spectrum of **B2-5**, Figure S3: ^1^H NMR spectrum of **B3-5**, Table S1: Crystallographic data of gold complexes, Figure S4: TG-DTA curve of all gold(I) complexes, Figure S5: DSC thermogram of **B2-4**, Figure S6: DSC thermogram of **B2-5**, Figure S7: DSC thermogram of **B3-5**; Figure S8: UV-Vis absorption, excitation, and emission spectra of **B2-4** and **B3-5** in CH_2_Cl_2_, Figure S9: AIE experiment of **B3-5**, Figure S10: emission spectra of **B3-5** in THF before and after degassing, Figure S11: photoluminescence decay profiles of gold(I) complexes.

## Sample Availability

Samples of the compounds are not available from the authors.
