# Supplementary material for: Aggregation-Enhanced Room-Temperature Phosphorescence from Au(I) Complexes Bearing Mesogenic Biphenylethynyl Ligands
Source: Molecules. 2021 Nov 30;26(23):7255. doi: 10.3390/molecules26237255 (PMC8658970; doi:10.3390/molecules26237255)
Supplement: Supplementary file 1 [file molecules-26-07255-s001.zip › molecules-1485148-supplementary.pdf]

molecules

MDPI

Supplementary materials

Aggregation-enhanced room-temperature phosphorescence from Au(I) complexes bearing mesogenic biphenylethynyl ligands

Andriani Furoida,[1] Misato Daitani,[1] Kyohei Hisano,[1] and Osamu Tsutsumi[1,*]

[1] Department of Applied Chemistry, Ritsumeikan University, 1-1-1 Nojihigashi, Kusatsu 525-8577, Japan
* Correspondence: tsutsumi@sk.ritsumei.ac.jp

Table of Contents

1. **^{1}H NMR specta** **S3**

2. **X-Ray crystallography data** **S4**

3. **Thermochemical Properties** **S6**

4. **Photophysical properties** **S8**

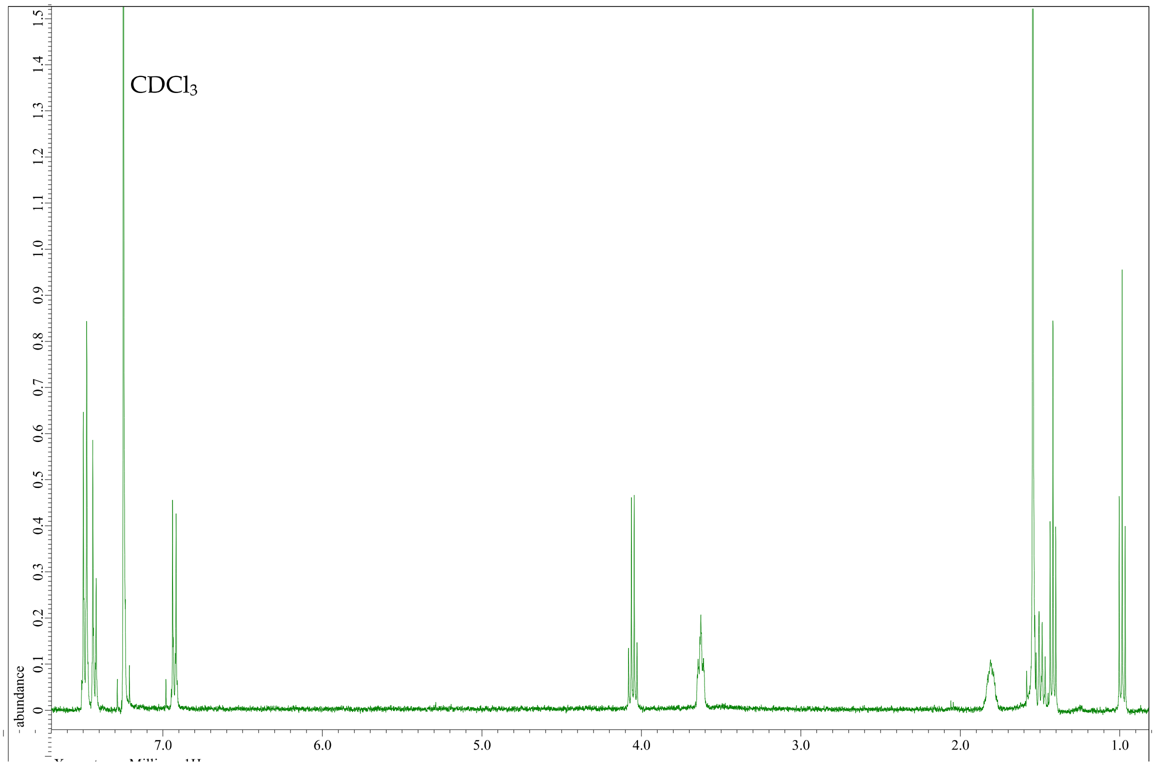

Figure S1. ¹H NMR spectra of **B2-4**.

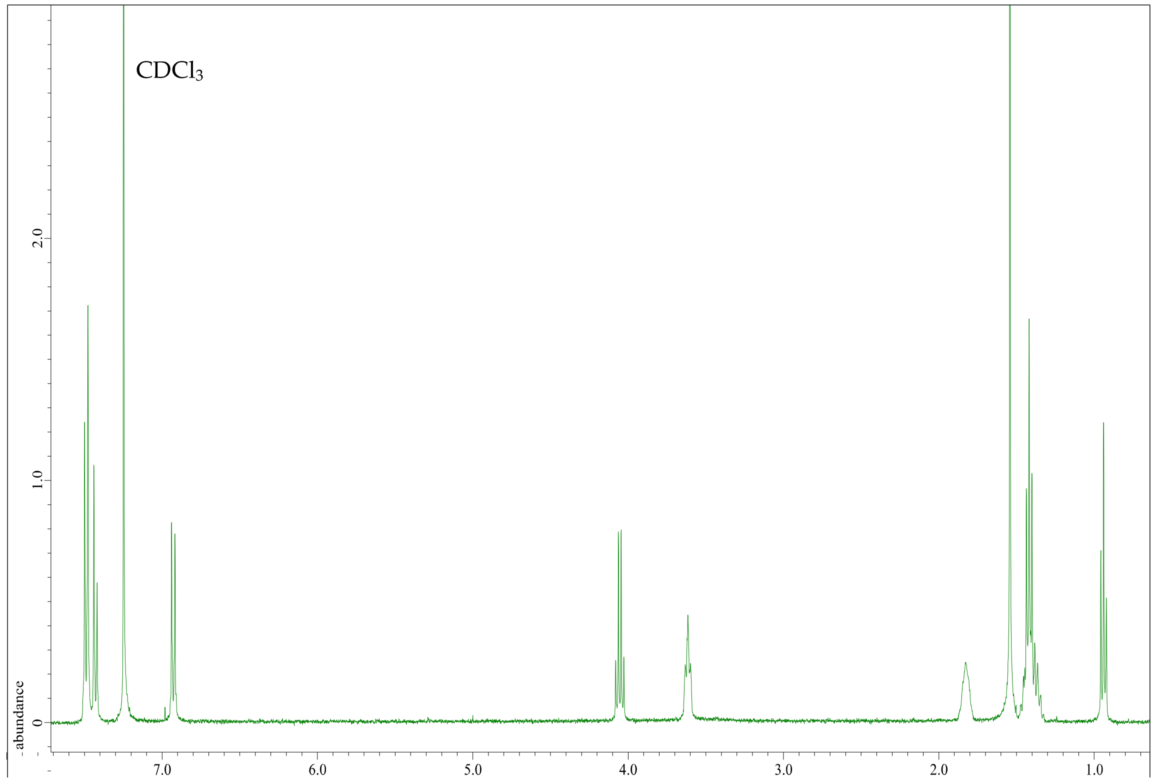

Figure S2. ¹H NMR spectra of **B2-5**.

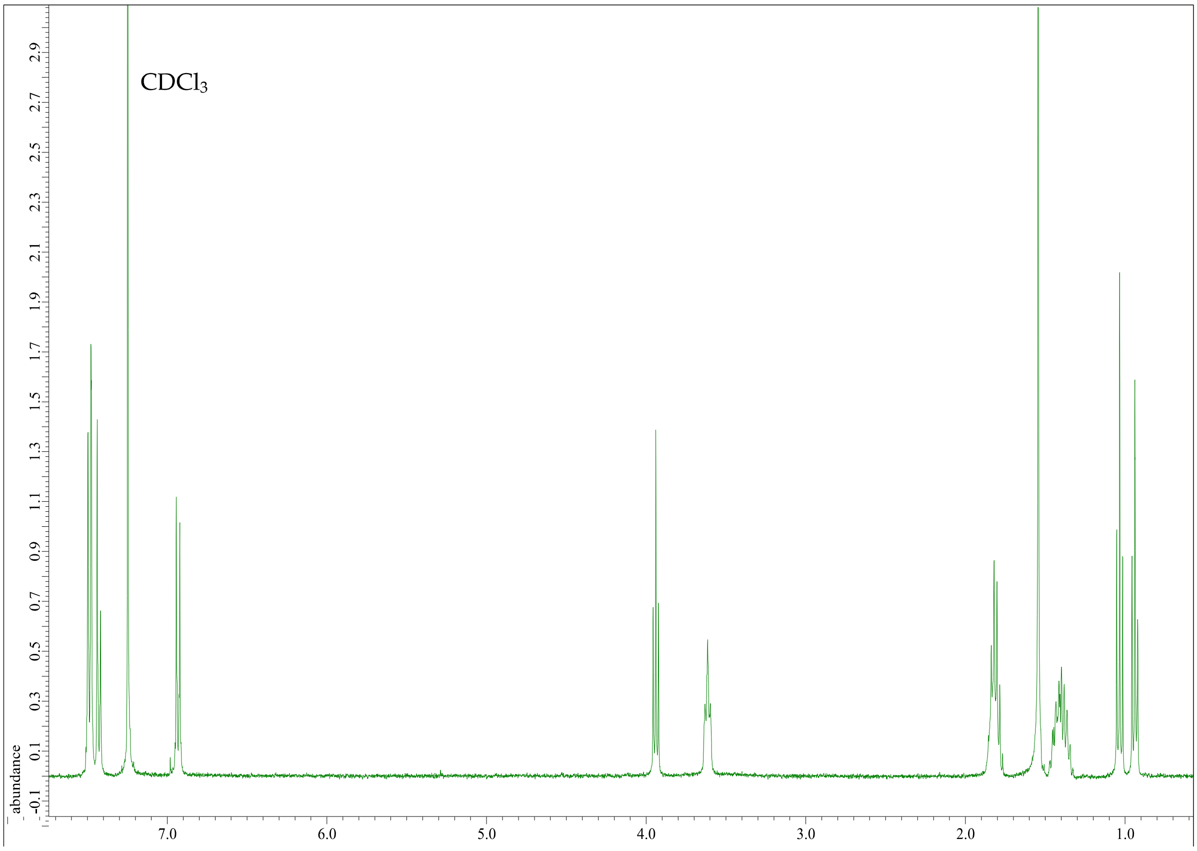

Figure S3. ^{1}H NMR spectra of **B3-5**.

Table S1. Crystallographic data of gold(I) complexes.

	**B2-4**	**B2-5**	**B3-5**
Radiation	Mo K*α*	Mo K*α*	Mo Kα
Empirical formula	$C_{21}H_{22}AuNO$	$C_{22}H_{24}AuNO$	$C_{23}H_{26}AuNO$
Formula weight	501.36	515.39	529.41
Temperature	293 K	293 K	293 K
Crystal color, Habit	yellow, prism	yellow, prism	yellow, needle
Crystal Dimensions (mm)	0.5 × 0.5 × 0.3	0.7 × 0.7 × 0.5	0.7 × 0.03 × 0.03
Crystal system	triclinic	triclinic	triclinic
$R[F^2>2\sigma(F^2)]$	0.0715,	0.0475,	0.0484,
$wR(F^2)$	0.1605	0.1281	0.1407
S	1.764	1.764	1.670
Space group	*P*-1	*P*-1	*P*-1
Z	4	2	2
a (Å)	10.5085 (15)	7.6775 (7)	8.1315 (4)
b (Å)	12.7427 (18)	9.4496 (9)	9.3466 (5)
c (Å)	15.0687 (18)	14.1786 (19)	15.5315 (8)
α (°)	110.690 (4)	106.381 (4)	76.587 (2)
β (°)	90.119 (5)	98.555 (5)	85.158 (2)
γ (°)	90.007 (5)	93.350 (4)	66.488 (2)
V (Å^3)	1887.7 (4)	970.43 (18)	1052.85 (10)

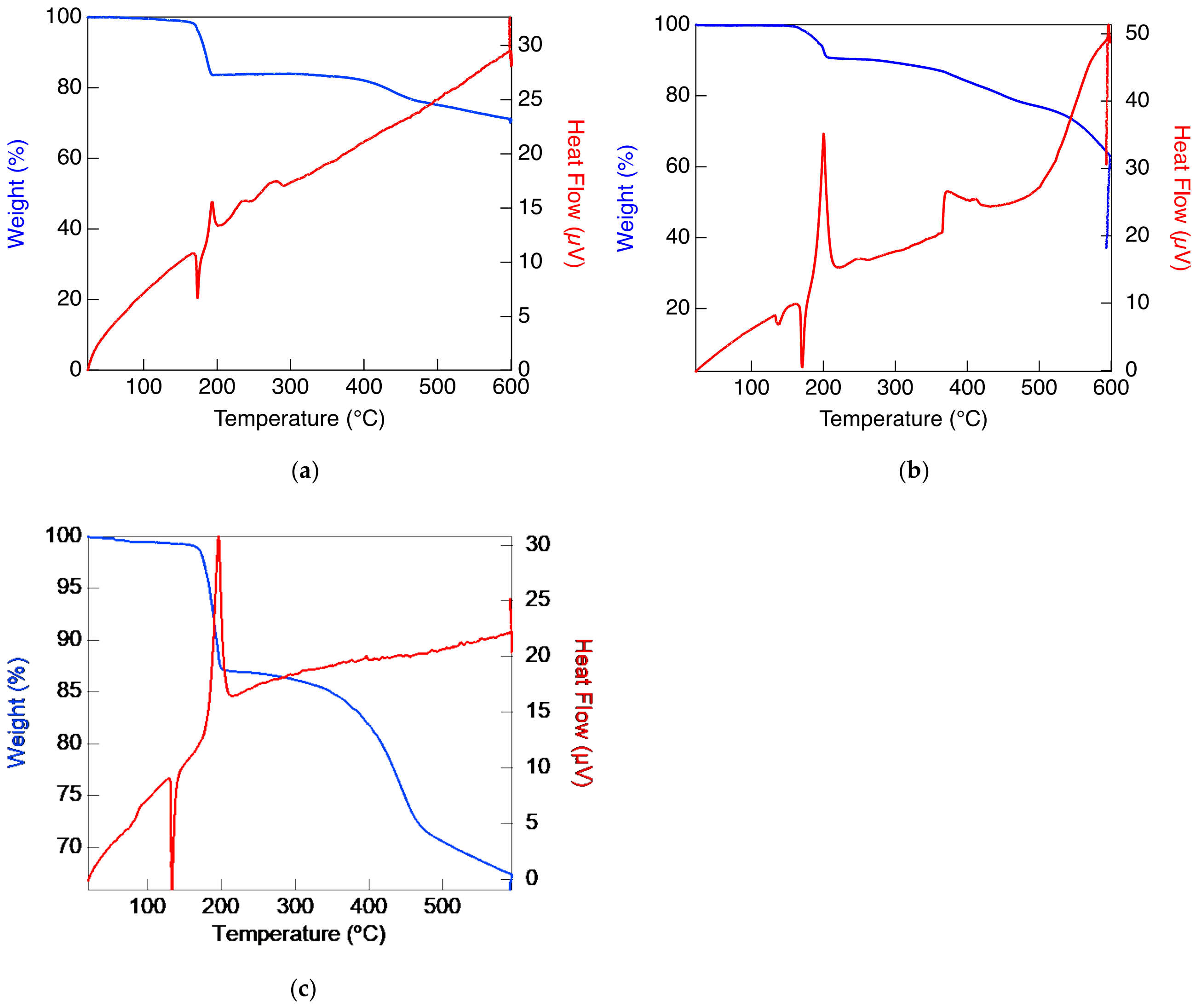

Figure S4. TG-DTA curves of (**a**) **B2-4**, (**b**) **B2-5**, and (**c**) **B3-5** (scanning rate, 5 °C/min; red, TGA curve; blue, DTA curve).

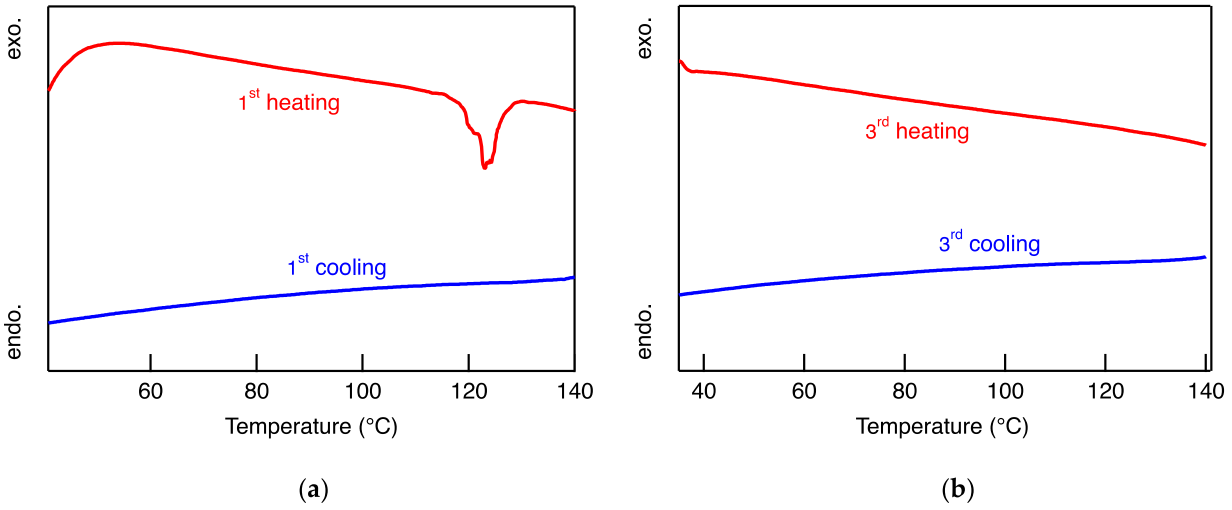

Figure S5. DSC thermograms of **B2-4** in the (**a**) first and (**b**) third scanning process (scanning rate, 5 °C/min; red, heating; blue, cooling).

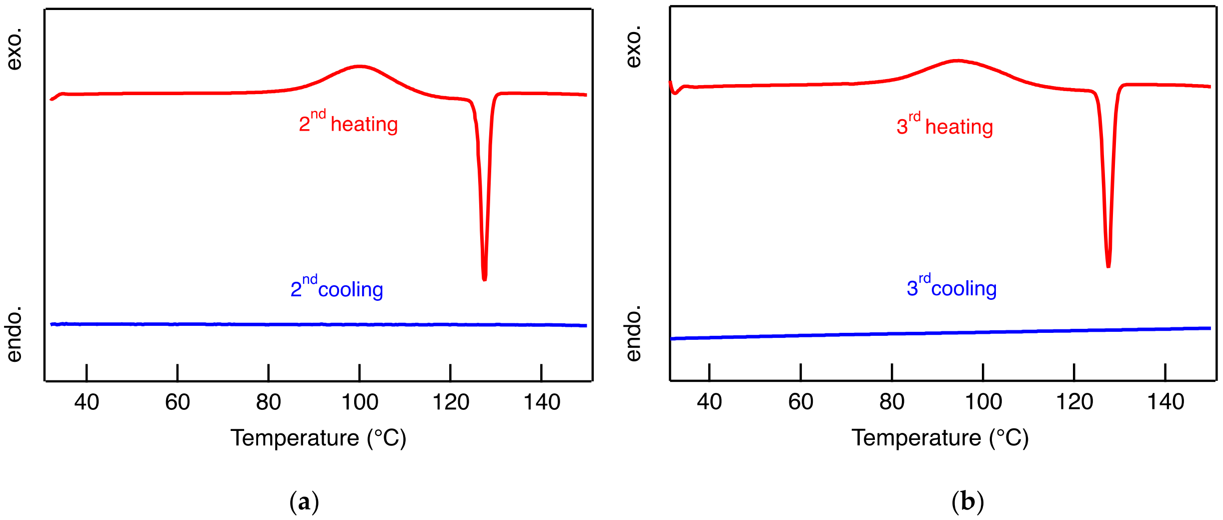

Figure S6. DSC thermograms of **B2-5** in the (**a**) second and (**b**) third heating-cooling process (scanning rate, 5 °C/min; red, heating; blue, cooling) and (scanning rate, 3 °C/min; red, heating; blue, cooling).

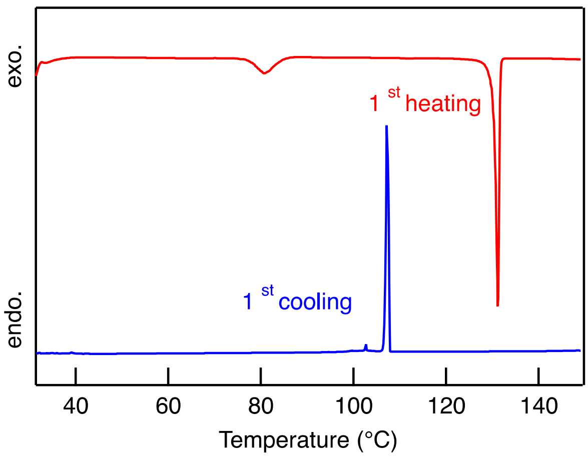

Figure S7. DSC thermograms of **B3-5** in the first heating-cooling process (scanning rate, 2 °C/min; red, heating; blue, cooling).

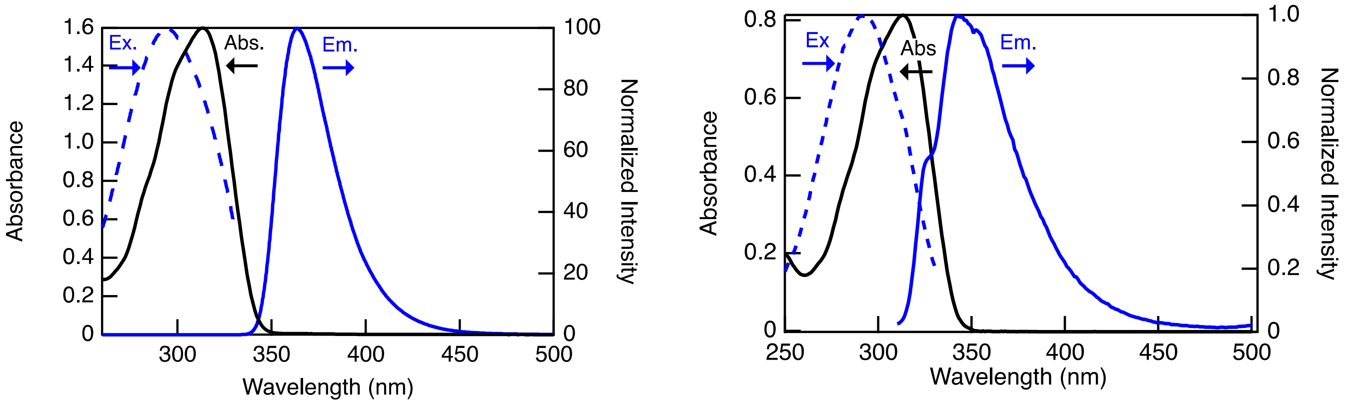

(**a**) (**b**)

Figure S8. Photophysical properties of (**a**) **B2-4** and (**b**) **B3-5** in CH_2Cl_2 solution: absorption, solid black line; emission, solid blue line; excitation, dashed blue line. Concentration used are 1 × 10^{-5} mol L^{-1} for absorption, and 1.5 × 10^{-6} mol L^{-1} for luminescence spectra. The excitation wavelength (λ_{ex}) for **B2-4** and **B3-5** were 294 nm and 291 nm, respectively. Excitation spectra were obtained at 366 nm for **B2-4** and 350 nm for **B3-5**.

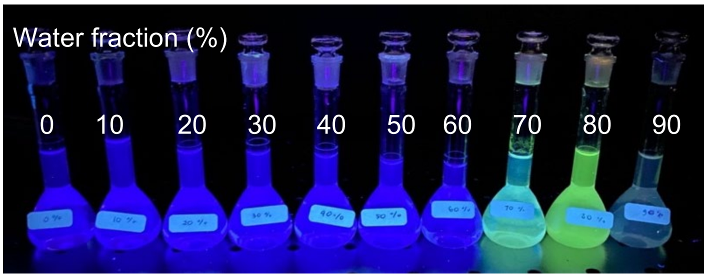

(**a**)

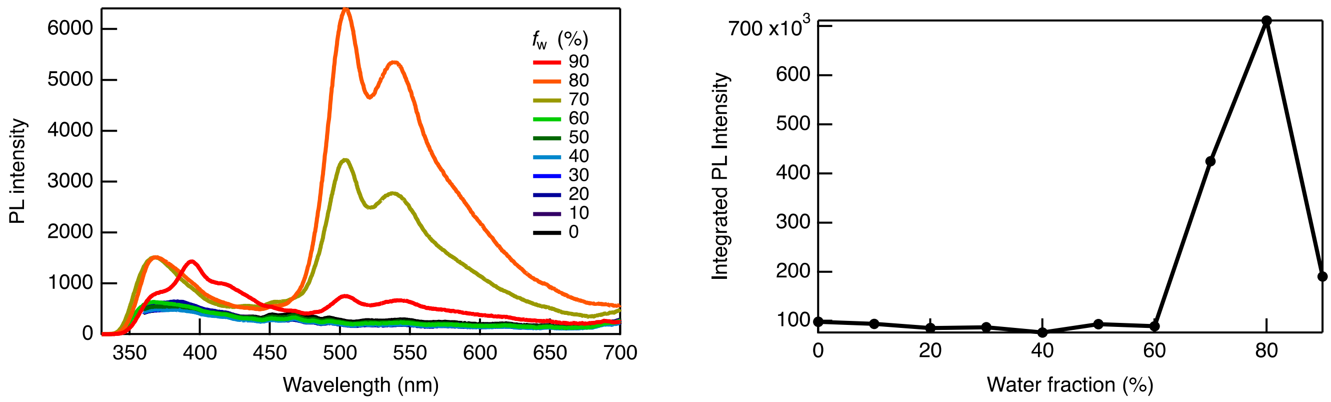

(**b**) (**c**)

Figure S9. (**a**) Photograph of **B3-5** in f_w = 0–90% under UV lamp at 365 nm, (**b**) Emission spectra of **B3-5** in f_w = 0–90% with concentration of 1 x 10^{-4} mol L^{-1} (λ_{ex} = 312 nm). (**c**) Integrated photoluminescence intensity in a range from 320 to 700 nm as a function of f_w.

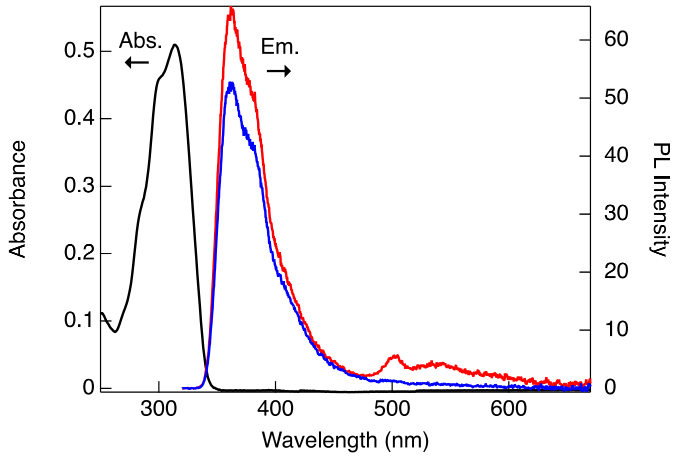

Figure S10. Photophysical properties of **B3-5** in THF solution. (black, absorption; blue, emission before degassing; red, emission after degassing). The excitation wavelength (λ_{ex}) was 280 nm. UV-Vis absorption concentrations: 1 × 10^{-5}, luminescence concentration: 1.5 × 10^{-6} mol L^{-1}.

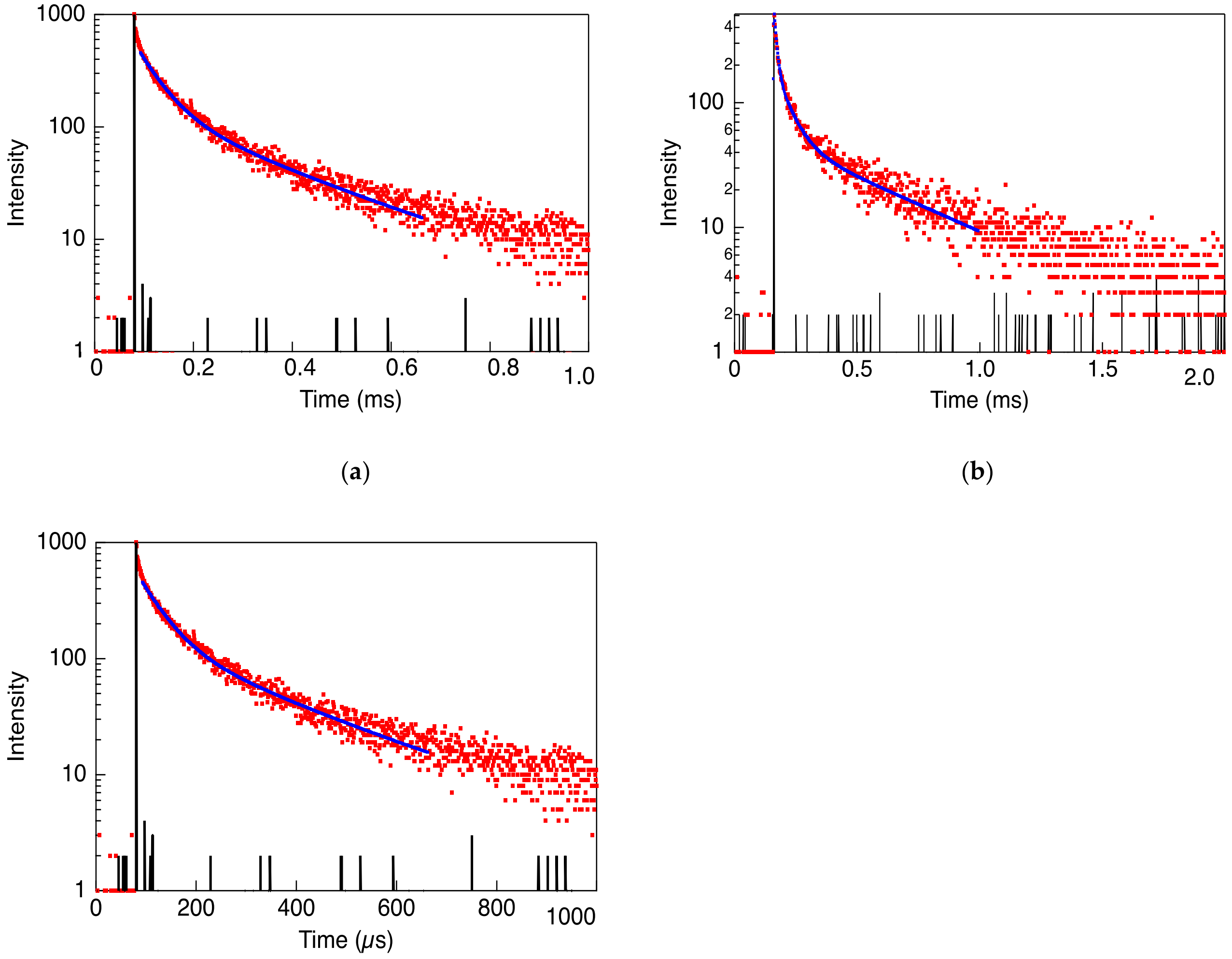

(**a**) (**b**)

(**c**)

Figure S11. Decay profiles of (**a**) **B2-4**, (**b**) **B2-5**, (**c**) **B3-5** crystal at 500 nm emission (λ_{ex} = 340 nm). Red, emission lifetime decay; black, IRF; blue, curve fitting.
